# Fermi surface topology and magnetotransport in semimetallic LuSb

**DOI:** 10.1038/s41598-017-12792-8

**Published:** 2017-10-09

**Authors:** Orest Pavlosiuk, Maja Kleinert, Przemysław Swatek, Dariusz Kaczorowski, Piotr Wiśniewski

**Affiliations:** 1 Institute of Low Temperature and Structure Research, Polish Academy of Sciences, P.Nr 1410, 50-950 Wrocław, Poland; 2 Present Address: Division of Materials Science and Engineering, Ames Laboratory, Ames, Iowa 50011 USA

## Abstract

Several rare-earth monopnictides were shown to exhibit extreme magnetoresistance and field-induced low-temperature plateau of electrical resistivity. These features are also hallmarks of topological semimetals, thus the family is intensively explored with respect to magneto-transport properties and possible hosting Dirac fermion states. We report a comprehensive investigation of Fermi surface and electrical transport properties of LuSb, another representative of this family. At low temperatures, the magnetoresistance of LuSb was found to exceed 3000% without saturation in fields up to 9 T. Analysis of the Hall effect and the Shubnikov–de Haas oscillations revealed that the Fermi surface of this compound consists of several pockets originating from fairly compensated multi-band electronic structure, in full accordance with our first-principles calculations. Observed magnetotransport properties of LuSb can be attributed to the topology of three-dimensional Fermi surface and a compensation of electron and hole contributions.

## Introduction

Rare-earth monopnictides have been studied intensively in the last two years as materials with extreme magnetoresistance (XMR), having potential in application as magnetic field sensors, but also bearing some similarities to topologically nontrivial semimetals. For LaSb, XMR = 9 × 10^5^% (at *T* = 2 K and *B* = 9 T) was reported^1^, comparable to XMR in archetypal Weyl semimetals TaAs and NbP^2,3^, and Dirac semimetal Cd_3_As_2_^4^. It has been suggested that XMR in this compound, alike in the bismuthide LaBi, emerges from nearly perfect carrier compensation and *d*−*p* mixed orbital texture of the Fermi surface (FS)^5^. The same reasoning was extended in ref.^5^ to other materials demonstrating XMR: NbSb_2_, PtSn_4_ and WTe_2_. Similarly large XMR effect was reported also for antiferromagnets: NdSb and CeSb^6,7^. Remarkably, cerium monopnictides were proposed recently as unique materials in which Dirac fermions coexist with strongly correlated electrons^8^. For both LaSb and LaBi, the authors of ref.^5^ emphasized a correlation between the magnitude of XMR and the square of the residual resistivity ratio (RRR = *ρ*(300K)/(2K)). Similar correlation was shown for these monopnictides over two decades ago^9^.

As far as topological character of the electronic structure in LaSb is concerned, there are distinctly controversial reports in the literature. The first angle-resolved photoemission spectroscopy (ARPES) study indicated trivial electronic structure and confirmed that unsaturated XMR is a consequence of nearly perfect compensation of charge carriers^10^. Shortly thereafter, another ARPES experiment revealed exotic surface states^11^, resembling those in LaBi, where non-trivial topology of the electronic structure was concluded from the results of electronic transport^12,13^ and ARPES measurements^14–16^. Very recent comprehensive Kohler scaling analysis clearly indicated the bulk origin of XMR in LaSb^17^.

Formation of lutetium monoantimonide was first reported more than half-century ago^18^. In early studies, this compound was used only as a non-magnetic reference or an initial matrix for doping with other rare-earths^19,20^. Later, some results of electronic transport, thermal expansion and micro-hardness measurements as well as melting point were reported^21^. Following discovery of the first-order structural phase transition at high pressures in rare-earth monoantimonides, among them LuSb^22^, stronger interest in this family of compounds was aroused, resulting in several theoretical analyses of electronic, elastic and structural properties^23–26^.

This work on LuSb, is a follow-up of our recent investigation of the magnetotransport properties of YSb, which is a well compensated semimetal with XMR attaining 1.6×10^4^% (at 2 K in magnetic field of 9 T)^27–29^. To date, single values of resistivity, carrier concentration and mobility of LuSb were available in the literature^21^, without specifying temperatures at which these quantities were obtained. In the very recent ARPES study no Dirac-like features were found^30^. In this paper we describe the magnetotransport properties and the Fermi surface of LuSb, and compare the experimental findings with the results of our electronic band structure calculations.

## Results and Discussion

### Electronic structure calculations

**Figure 1:**
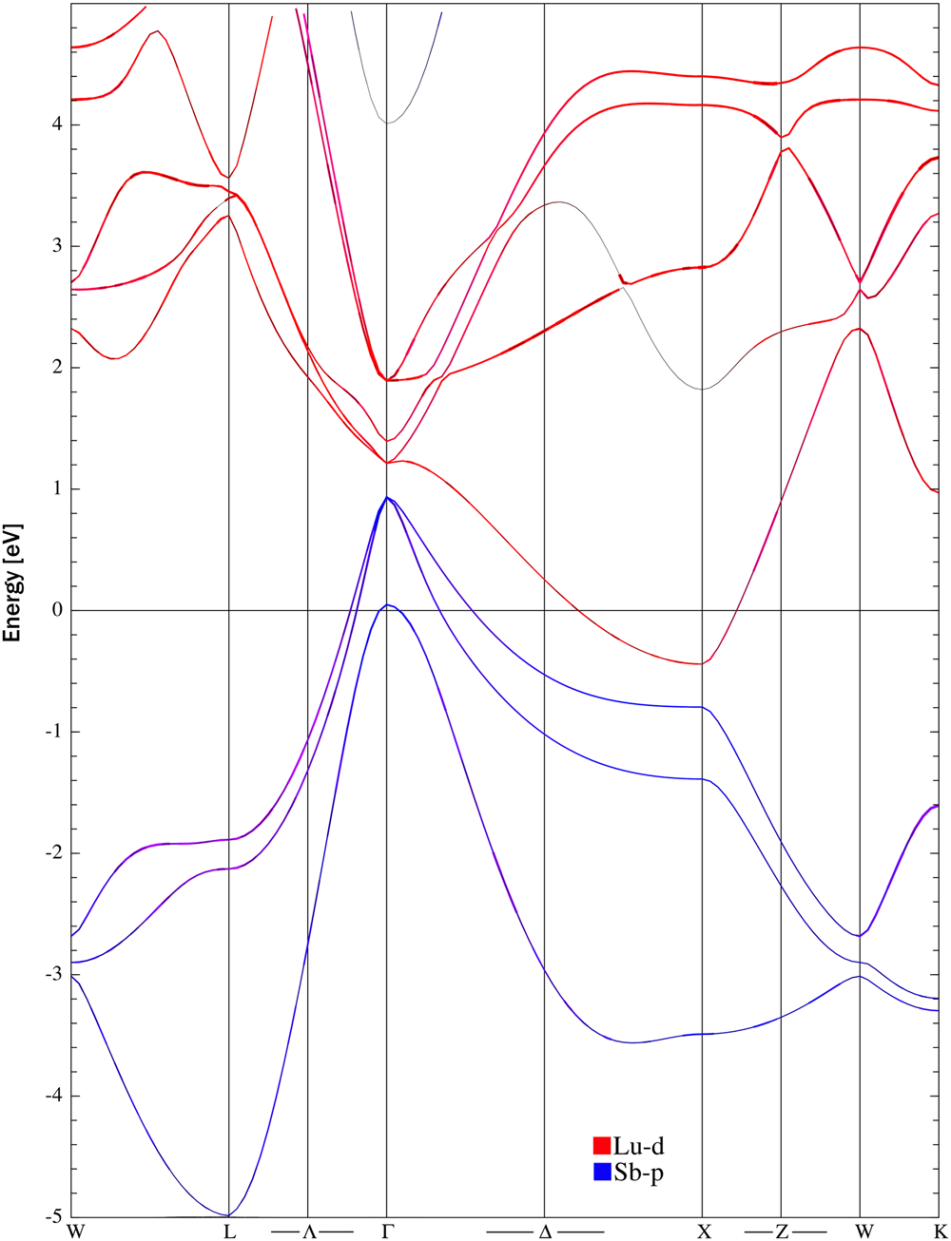
Electronic band structure of LuSb. Red and blue mark the bands originating from Lu-*d* and Sb-*p* electrons, respectively.

Figure 1 presents the electronic structure of LuSb calculated using LAPW method with the modified Becke-Johnson (mB-J) potential (details are described in Methods). Overall, it is very similar to those reported for other rare-earth monopnictides^5,12,13,27,31^, with four bands crossing the Fermi level: three hole bands in vicinity of Γ-point and one electron band close to the high-symmetry X-point of the Brillouin zone.

The *d* − *p* mixed orbital texture in LaSb and LaBi is due to the crossing of the lanthanum *d*-band with the pnictogen *p*-band and subsequent opening of a small gap at the point of crossing by the spin-orbit interaction. This was suggested to be responsible for topological nature of these compounds^5,31^. We reported very similar texture in YSb^27^.

In LuSb, however, the *d*-band of lanthanum and the *p*-band of antimony do not cross between Γ and X points, but are well separated by a direct gap of at least 0.38 eV (cf Fig. 1). The gaps opened by the spin-orbit coupling in lanthanum monopnictides^5,31^ and YSb^27^ were two or three orders of magnitude narrower than that in LuSb. These features exclude any significant *d* − *p* orbital mixing, which could give rise to topologically non-trivial electronic states in this compound.

**Figure 2:**
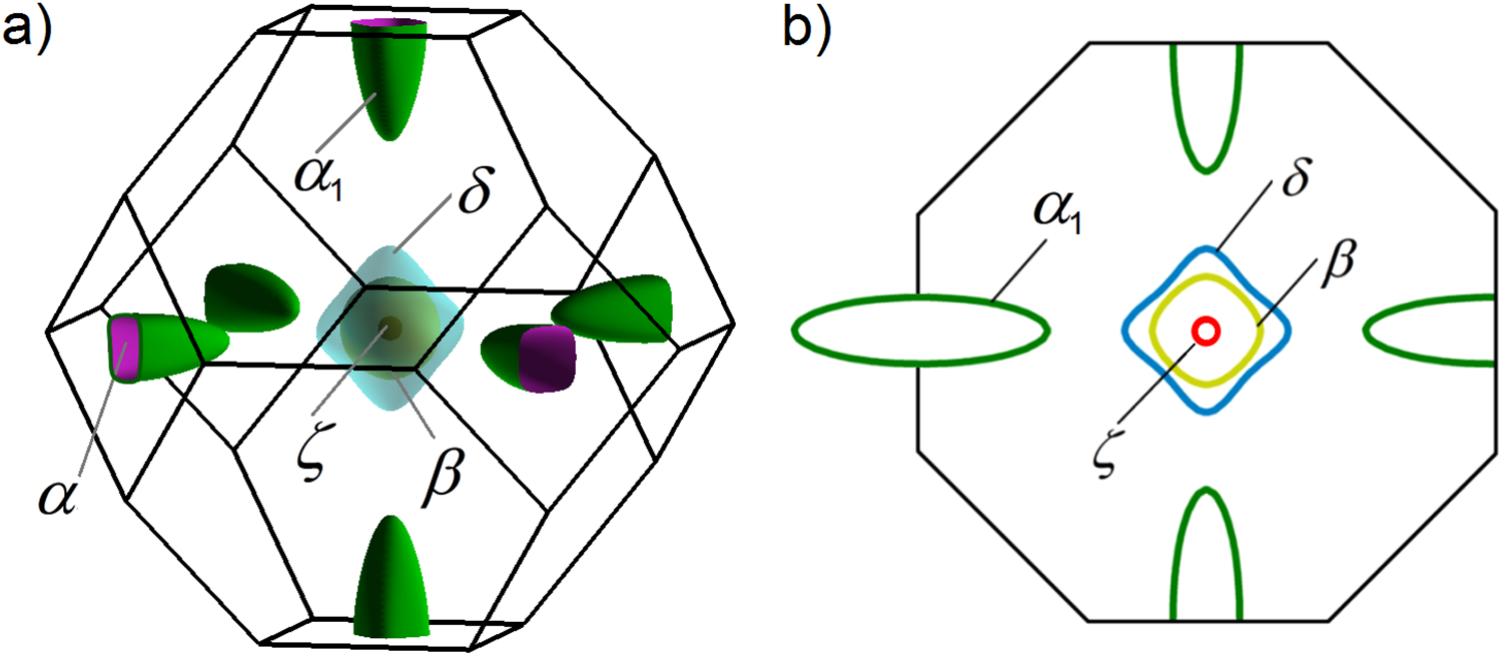
(**a**) Fermi surface of LuSb in the first Brillouin zone consists of a triplicate electron pocket (*α*) and three hole pockets (*β*, *δ* and *ζ*). (**b**) Cross-section of the Brillouin zone with (001) plane passing through its central Γ-point.

The calculations showed that the FS of LuSb consists of four sheets (see Fig. 2). Three of them, centered at the Γ-point (labeled *β*, *δ*,* ζ* in Fig. 2(a)), are hole-like, whereas a triplicate one, centered at the X-point (labeled *α* in Fig. 2(a)), is electron-like. The smallest *ζ* pocket is a sphere placed inside the almost spherical *β* pocket, which in turn, is nested in the biggest hole-pocket *δ*, resembling an octahedron.

FS resulting from our calculations differs slightly from those reported before, which did not show the smallest hole-like pocket^24,25^. The Fermi surfaces calculated for lanthanum monopnictides are also different, having two hole-like Fermi sheets^5,13^. However, the data derived for LuSb are almost identical to those of YSb^27^. Comparing total volume of three hole pockets (0.06967 Å^−3^) with that of the electron pocket (0.07065 Å^−3^) leads to a conclusion that LuSb is fairly well compensated semimetal.

### Electrical resistivity and magnetoresistance

**Figure 3:**
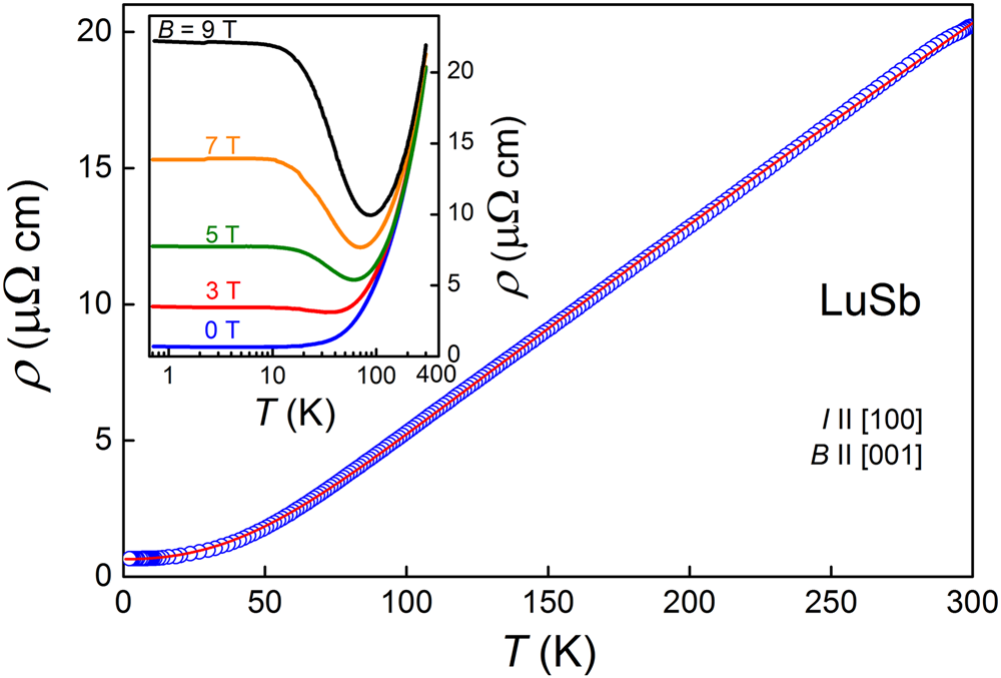
Electrical resistivity of LuSb as a function of temperature. Red solid line corresponds to the fit with Bloch-Grüneisen formula (equation 1). Inset: temperature variations of the electrical resistivity of LuSb recorded in several magnetic fields applied perpendicular to the electric current.

As shown in Fig. 3, the electrical resistivity of single-crystalline LuSb decreases with decreasing temperature in a metallic-like manner from *ρ* = 201.9 *μ*Ω cm at *T* = 300 K down to *ρ* = 0.67*μ*Ω cm at *T* = 2 K. The RRR of our sample is 30, slightly larger than 22, we reported for YSb^27^, but about tenfold smaller than those of several other rare-earth monopnictides^1,11–13^. In the temperature interval from 0.7 K to 300 K, *ρ*(*T*) follows the Bloch-Grüneisen law:

1 $$\rho (T)={\rho }_{0}+A{(\frac{T}{{{\rm{\Theta }}}_{D}})}^{k}{\int }_{0}^{\frac{{{\rm{\Theta }}}_{D}}{T}}\frac{{x}^{k}}{({e}^{x}-\mathrm{1)(1}-{e}^{-x})}dx,$$

with the residual resistivity *ρ*_0_ = 0.65 *μ*Ω cm, Debye temperature ${{\rm{\Theta }}}_{D}=408$ K, factor *A* = 33.7 *μ*Ω cm and exponent *k* = 2.19 (note red solid line in Fig. 3). The obtained value of *k* is similar to those observed for several La- or Lu-intermetallics^32^, and another lutetium monopnictide, LuAs, which has also a very similar ${{\rm{\Theta }}}_{D}=420$ K^33^.

In magnetic fields applied perpendicular to the electric current, the electrical transport in LuSb has a completely different character than that in zero magnetic field (see the inset to Fig. 3). In *B* = 3 T, a shallow minimum in *ρ*(*T*) can be discerned at *T*_*m*_ ≈ 34 K, and in stronger fields this feature becomes more pronounced and shifts to higher temperatures (*T*_*m*_ ≈ 88 *K* in 9 T field). Below *T*_*m*_, the resistivity increases with decreasing *T*, and saturates below ≈10 K, forming a low-temperature plateau. Such a behavior of *ρ*(*T*) was described as a characteristic feature of topological semimetals^3^, or alternatively, attributed to band compensation effect occurring e.g. in WTe_2_^34^ or several rare-earth monopnictides^12,13,27^.

Universal *T* − *B* phase diagram was constructed for several materials showing XMR (LaSb, LaBi, NbSb_2_, PtSn_4_ and WTe_2_)^5,13^. It consisted of linear-with-field *T*_*m*_ and field-independent *T*_*i*_ (marking the inflection point of *ρ*(*T*)). The *T*_*m*_ and *T*_*i*_ temperatures discerned from the inset to Fig. 3 also form a similar phase diagram for LuSb.

**Figure 4:**
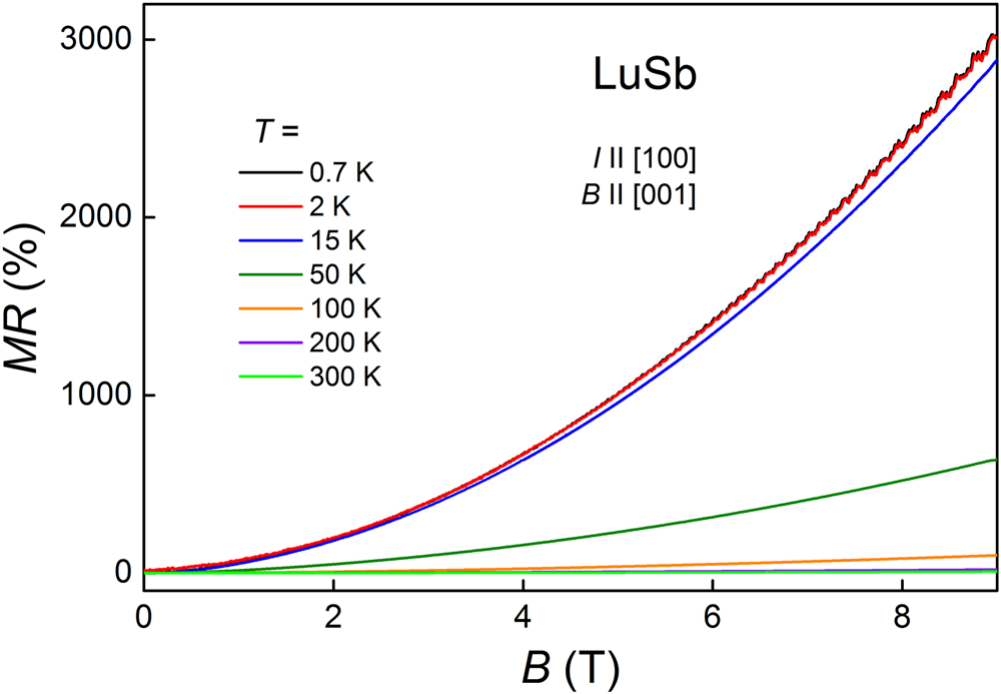
Isotherms of the transverse magnetoresistance of LuSb versus magnetic field.

The transverse magnetoresistance of LuSb, MR = $100{\rm{ \% }}\times [\rho (B)-\rho (B=0)]/\rho (B=0)$ (magnetic field applied perpendicular to electric current), measured at several temperatures in the range from 0.7 K up to 300 K, is displayed in Fig. 4. MR reaches 3025% at *T* = 0.7 K, and MR(*B*) does not saturate in a field of 9 T. Upon heating the sample up to 15 K, MR decreases very little, but between 15 and 50 K it drops drastically. The magnetoresistance of LuSb is one or two orders of magnitude smaller that MR reported for other rare-earth monopnictides^5,12,13,29^. This seems due to rather small RRR of our crystals, since it was shown that MR depends on RRR in a quadratic manner^5,35^. In previous works, XMR of rare-earth monopnictides was attributed to: almost perfect electron-hole compensation^12,13,29^, metal-insulator transition^1^ or *d*−*p* orbital mixing combined with carrier compensation^5^. The last scenario was also proposed for some other materials with XMR effect, like WTe_2_, NbSb_2_, or PtSn_4_^5^. We suggest that electron-hole compensation leads to very large MR in LuSb.

**Figure 5:**
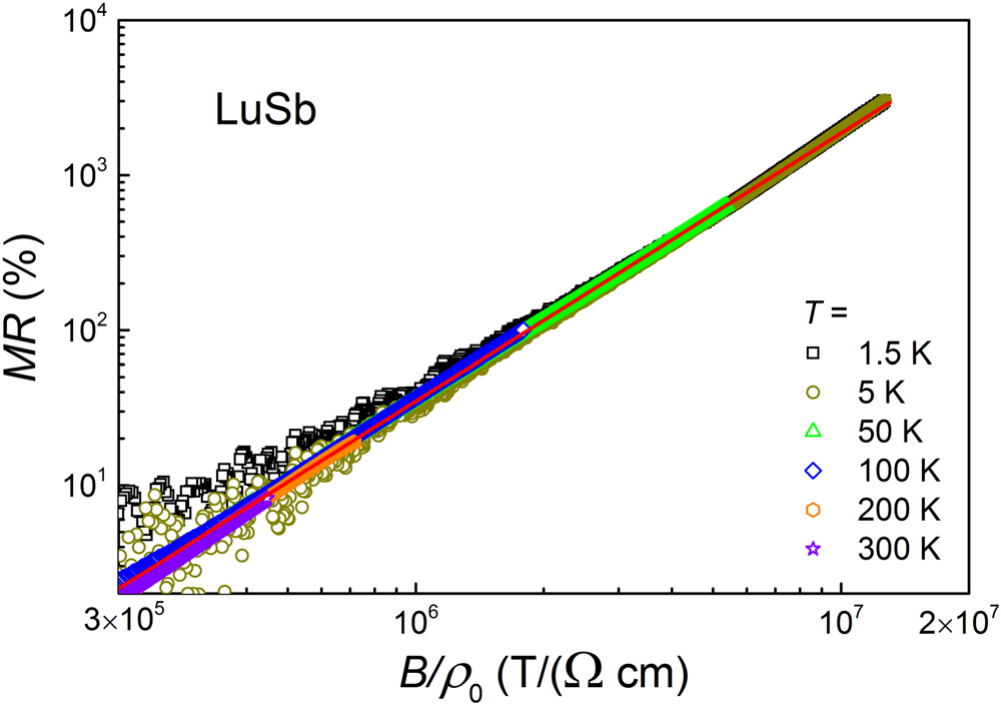
Kohler scaling of the magnetoresistance of LuSb. Red line corresponds to the fit with Kohler’s equation.

The Kohler scaling, which is presented in Fig. 5, allows to draw conclusions about the very large MR effect. For LuSb, all the MR curves measured at different temperatures collapse onto a single curve that can be described by the Kohler equation MR$=\alpha {(B/\rho (B=\mathrm{0))}}^{m}$ with the exponent *m* = 1.73 (note a red solid line in Fig. 5). The obtained value of *m* is nearly the same as that derived for YSb^27^ but somewhat smaller than *m* = 1.866 and *m* = 1.92 reported for LaBi^13^, and WTe_2_^34^, respectively. It is worth recalling that *m* = 2 is expected for a material with perfect carrier compensation.

**Figure 6:**
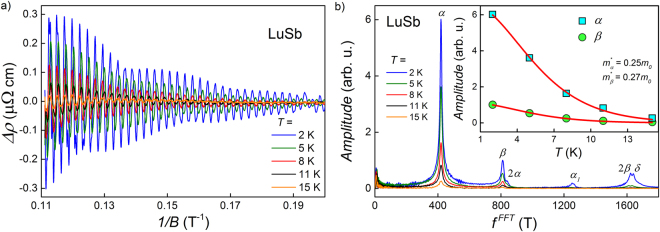
(**a**) Oscillating part of the electrical resistivity of LuSb plotted versus inverse magnetic field for several temperatures. (**b**) Fast Fourier transform spectra obtained for SdH oscillations shown in panel (a). Inset: temperature dependence of the amplitudes of two main peaks in the FFT spectra. Solid lines represent fits of equation (2) to the experimental data.

In magnetic fields stronger than ~5 T, the transverse magnetoresistance of LuSb shows clear Shubnikov–de Haas (SdH) oscillations at temperatures up to 15 K (cf Fig. 4). The result of subtraction of a 2^nd^-order-polynomial background from the resistivity data, Δ*ρ*, is plotted in Fig. 6(a) against inverse magnetic field for several temperatures from the range 2–15 K. Fast Fourier transform (FFT) analysis (see Fig. 6(b)), performed on the Δ*ρ*(1/*B*) data, revealed that the observed oscillations contain four basic frequencies and two harmonics (the values of ${f}_{i}^{{\rm{FFT}}}$ are listed in Table 1). Multifrequency quantum oscillations clearly indicate complex Fermi surface structure of LuSb, in accord with the results of the electronic structure calculations presented above.

**Table 1:** Parameters obtained from the FFT analysis of SdH oscillations in LuSb recorded at *T* = 2 K. Particular symbols are described in the text. The value of ${k}_{F,{\alpha }_{1}}$ was estimated for direction of the longest axis of approximate ellipsoid. The ${f}_{i}^{calc}$ frequencies obtained from the LAPW band-structure calculations (adjusted by a factor 0.855, as explained in the text) are shown for comparison, together with corresponding carrier concentrations ${n}_{i}^{calc}$.

*i*=		*α*	*α*_1_	2*α*	*β*	2*β*	*δ*
${f}_{i}^{{\rm{FFT}}}$	(T)	420	1250	840	810	1620	1640
*k*_*F,i*_	(Å^−1^)	0.113	0.336	—	0.157	—	0.223
*n*_*i*_	(cm^−3^)	4.35 × 10^20^		—	1.31 × 10^20^	—	3.76 × 10^20^
${f}_{i}^{calc}$	(T)	410	1260	—	810	—	1640
${n}_{i}^{calc}$	(cm^−3^)	5.69 × 10^20^		—	1.65×10^20^	—	3.94 × 10^20^

According to Onsager relation^36^, oscillation frequencies are proportional to areas of extremal cross-sections of Fermi surface pockets. Thus, one can compare the experimental values of ${f}_{i}^{{\rm{FFT}}}$ with those estimated from the first-principle calculations, and ascribe each of them to a Fermi pocket. In the FFT spectrum of LuSb two different frequencies, ${f}_{\alpha }^{{\rm{FFT}}}$ and ${f}_{{\alpha }_{1}}^{{\rm{FFT}}}$ correspond to two different extreme cross-sections of the Fermi sheet *α* (marked as green loops in Fig. 2(a,b)). In turn, the frequencies ${f}_{\beta }^{{\rm{FFT}}}$ and ${f}_{\delta }^{{\rm{FFT}}}$ correspond to Fermi pockets *β* and *δ*, respectively (note blue and yellow loops in Fig. 2(b)). The frequency ${f}_{2\alpha }^{{\rm{FFT}}}$ is the second harmonic of ${f}_{\alpha }^{{\rm{FFT}}}$, while ${f}_{2\beta }^{{\rm{FFT}}}$ is the second harmonic of ${f}_{\beta }^{{\rm{FFT}}}$. We could not observe the contribution due to the smallest *ζ* Fermi sheet because the range of magnetic field in which we recorded oscillations was too narrow to detect such a small frequency as ${f}_{\zeta }\approx 50$ T predicted by calculations.

Making rough approximations that extreme cross-sections of *α*, *β* and *δ* are circles, and extreme cut of *α*_1_ is an ellipse (see Fig. 2), we calculated the Fermi wave-vectors, *k*_*F*,*i*_, gathered in Table 1. Subsequently, we calculated volumes, *V*_*F*,*i*_), and corresponding carrier concentrations, ${n}_{i}={V}_{F,i}/\mathrm{(4}{\pi }^{3})$, for each Fermi pocket. The so-obtained values of *n*_*i*_ are given in Table 1 (the value *n*_*α*_ takes into account the triplicity of the *α* Fermi pocket).

In the inset to Fig. 6, the amplitudes of peaks corresponding to the *α* and *β* Fermi pockets are plotted as a function of temperature. From the least-square fitting with the equation describing thermal damping of SdH oscillation

2 $${R}_{i}(T)=(\lambda {m}_{i}^{\ast }T/B)/\sinh (\lambda {m}_{i}^{\ast }T/B),$$

with *B* = 9 T and the constant $\lambda =2{\pi }^{2}{k}_{B}{m}_{0}/e\hslash \,(\approx 14.7\,{\rm{T}}/{\rm{K}})$, we obtained the effective masses: ${m}_{\alpha }^{\ast }=0.25\,{m}_{0}$ and ${m}_{\beta }^{\ast }=0.27\,{m}_{0}$. These values of *m* ^*^ are similar to those reported in the literature for other rare-earth monopnictides^6,12,13,27^.

**Figure 7:**
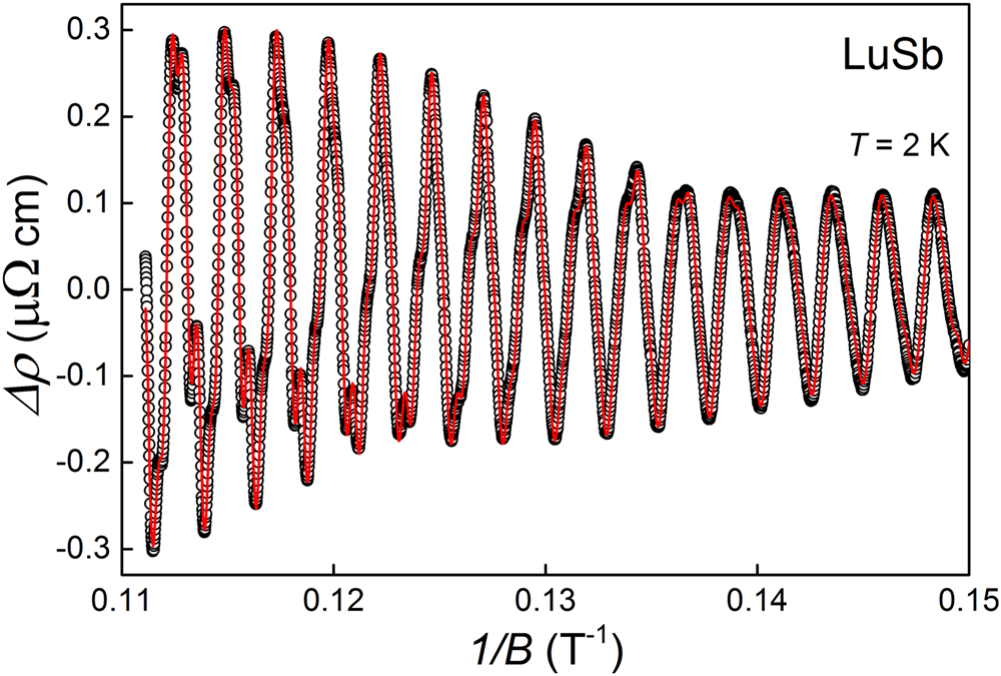
Oscillating part of the electrical resistivity of LuSb measured at *T* = 2 K, plotted versus inverted magnetic field. Red solid line represents the fit with equation (3).

In order to corroborate the FFT results, we evaluated the SdH oscillations in LuSb in terms of Lifshitz-Kosevich (LK) theory^36–38^. The multi-frequency LK function:

3 $${\rm{\Delta }}\rho =\sum _{i}{a}_{i}\sqrt{1/B}\,\frac{\exp (-{c}_{i}/B)}{\sinh ({b}_{i}/B)}\,\cos (2\pi ({f}_{i}/B-{\phi }_{i}-\frac{1}{8}));\,(i=\alpha ,{\alpha }_{1},\,2\alpha ,\beta ,\,2\beta ,\delta ),$$

was fitted to the experimental data taken at *T* = 2 K where, *f*_*i*_ is the oscillation frequency, ${a}_{i}\sqrt{1/B}{\sinh }^{-1}({b}_{i}/B)$ comprises the temperature reduction and the spin factors, $\exp (-{c}_{i}/B)$ stands for the Dingle factor and ${\phi }_{i}$ is the phase of oscillation. In order to avoid over-parametrization all frequencies were fixed at FFT-derived values (cf Table 1). The red fitting curve almost perfectly follows experimental ${\rm{\Delta }}\rho \mathrm{(1}/B)$ data, as shown in Fig. 7. This confirms that our FFT analysis revealed all significant contributions to SdH oscillations.

### Hall effect

**Figure 8:**
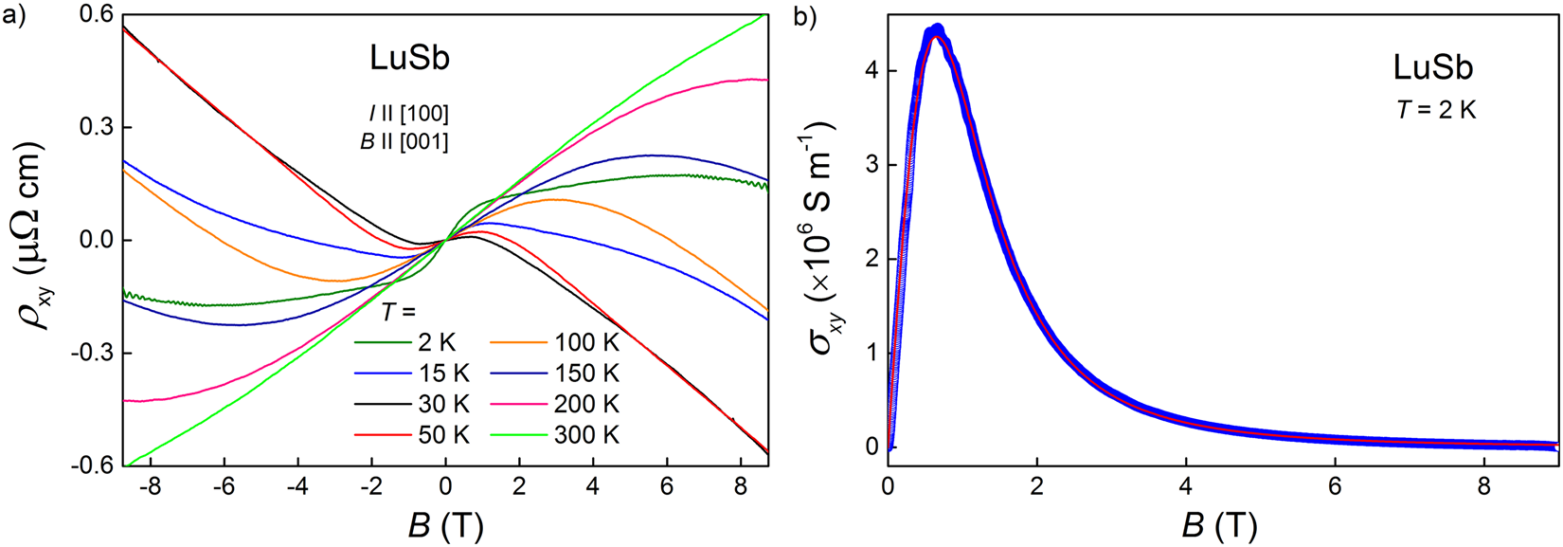
(**a**) Field variations of the Hall resistivity of LuSb measured at several different temperatures. (**b**) Hall conductivity versus magnetic field taken for LuSb at *T* = 2 K. Red line represents the fit with equation (4).

The results of Hall effect measurements of single-crystalline LuSb are summarized in Fig. 8. The Hall resistivity isotherms shown in Fig. 8(a) were obtained after removing the *ρ*_*xx*_ contribution by subtraction of the data recorded in magnetic fields of opposite directions. The magnitude of *ρ*_*xy*_ is very small and does not exceed 0.6 *μ*Ω cm for any field or temperature values accessible in the experiment. The *ρ*_*xy*_(*B*) dependences are curvilinear and nonmonotonous, which indicates that several bands are responsible for the transport properties of LuSb, in agreement with the results of the first-principle calculations and the SdH oscillations analysis presented above.

Accordingly, the Hall-effect data of LuSb were evaluated within a multi-band Drude model of electrical conductivity. First, *ρ*_*xy*_ was converted into the ${\sigma }_{xy}$ component of conductivity tensor using the simple relation ${\sigma }_{xy}=-{\rho }_{xy}/({\rho }_{xx}^{2}+{\rho }_{xy}^{2})$. Next, the ${\sigma }_{xy}(B)$ data were fitted with the multi-band Drude formula:

4 $${\sigma }_{xy}(B)=eB(\frac{-{n}_{el}{\mu }_{el}^{2}}{1+{({\mu }_{el}B)}^{2}}+\sum _{i=\,h\mathrm{1,}\,h2}\frac{{n}_{i}{\mu }_{i}^{2}}{1+{({\mu }_{i}B)}^{2}}),$$

where *e* is an elementary charge, *n*_*el*_ and *μ*_*el*_, *n*_*i*_ and *μ*_*i*_ (for $i=h1,h2$) stand for the carrier concentrations and the carrier mobilities of one electron- and two hole-like bands, respectively. Such three bands give main contributions to the conductivity of LuSb, as concluded from the analysis of the SdH oscillations.

Since the initial fit using free *n*_*el*_, *μ*_*el*_, *n*_*i*_ and *μ*_*i*_ parameters was over-parameterized, in the next step we fixed ${n}_{h1}$ at the carrier concentration value obtained from the SdH oscillations for the *β* pocket (the almost isotropic one) ${n}_{h1}\equiv {n}_{\beta }=1.31\times {10}^{20}$ cm^−3^. With this simplification, the experimental ${\sigma }_{xy}(B)$ data measured at *T* = 2 K were properly described with equation (4), as shown in Fig. 8(b) by a red solid line. The fit confirmed that two of three bands are hole-like and the third one is electron-like, and yielded the following parameters: ${\mu }_{h1}=1.04\times {10}^{4}$ cm^2^/(Vs), ${n}_{h2}=3.63\times {10}^{20}$ cm^−3^, ${\mu }_{h2}=6.18\times {10}^{3}$ cm^2^/(Vs), ${n}_{el}=4.93\times {10}^{20}$ cm^−3^ and ${\mu }_{el}=6.43\times {10}^{3}$ cm^2^/(Vs). It is worth noting that this analysis gave the ${n}_{h2}$ concentration almost identical to ${n}_{\delta }$ and the ${n}_{el}$ value intermediate between ${n}_{\alpha }$ and ${n}_{\alpha }^{calc}$ obtained from the SdH oscillations and band-structure calculations, respectively (cf Table 1). Small discrepancies may originate from the approximations of the *α* and $\delta$ Fermi sheets in LuSb with an ellipsoid and a sphere, respectively. On the other hand, our band-structure calculations were performed using lattice parameter obtained from X-ray diffraction at room temperature, slightly larger than at *T* = 2 K (thermal expansion of LuSb was predicted to be similar to that of normal metals^26^), which certainly influenced ${n}_{i}^{calc}$ values.

The sum of carrier concentrations of two hole-like bands ${n}_{h1}+{n}_{h2}\approx 4.94\times {10}^{20}$ cm^−3^ is almost equal to the carrier concentration *n*_*el*_ of the electron-like band. This indicates again that LuSb is close to perfect carrier compensation. In turn, the carrier mobilities in LuSb are comparable to those found for YSb^27^, yet an order of magnitude smaller than those reported for LaBi^12,13^. The moderate values of *μ*_*i*_ imply that the magnetoresistance of LuSb is notably smaller than MR measured for other rare-earth monopnictides^35^.

### Angle-dependent magnetotransport

**Figure 9:**
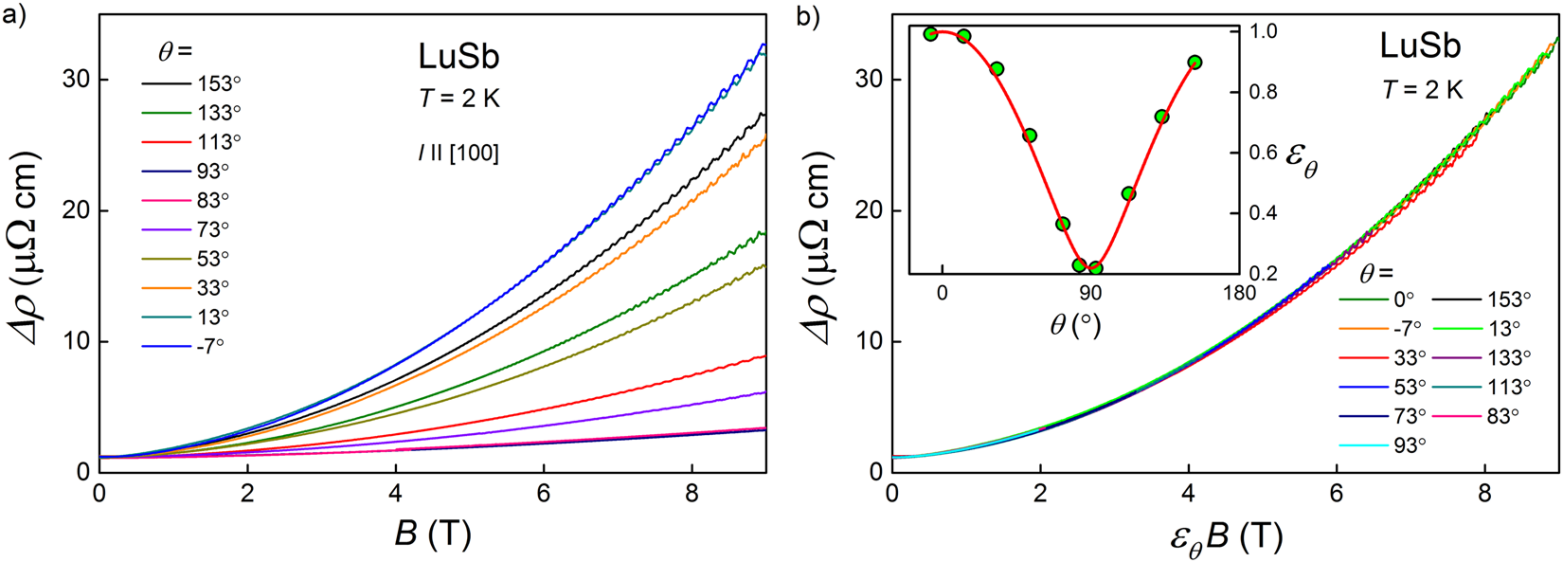
(**a**) Electrical resistivity of LuSb measured at *T* = 2 K as a function of magnetic field applied at different angles to the current direction, *θ*. (**b**) The electrical resistivity data from panel (a) plotted as a function of magnetic field scaled by factor ${\varepsilon }_{\theta }$. Inset: ${\varepsilon }_{\theta }$ as a function of angle *θ*. Red line represents the fit with equation (5).

Figure 9(a) illustrates changes in the field-dependent electrical resistivity of LuSb with varying the direction of applied magnetic field with respect to the direction of electric current. In a transverse configuration (field direction is perpendicular to current direction, which corresponds to *θ* = 0°), MR reaches its maximum magnitude, whereas in longitudinal configuration (field and current directions are parallel to each other, i.e. *θ* = 90°) MR attains its minimum values. It should be noted that the anisotropy of the magnetoresistance of LuSb (at *T* = 2 K and in *B* = 9 T) is giant and equals AMR $\equiv \,100{\rm{ \% }}\times [\rho {(90}^{\circ })-\rho {(0}^{\circ })]/\rho {(0}^{\circ })=-87\,{\rm{ \% }}$. This value is slightly larger than that observed for YSb^27^. In both compounds, AMR arises mainly due to carriers from the strongly anisotropic electron-like Fermi pockets.

In order to quantify the anisotropy of the Fermi surface in LuSb, the model developed in ref.^39^ was applied. First, the magnetic field was scaled by a *θ*-dependent factor ${\varepsilon }_{\theta }$, so as all the $\rho (B=9\,T)$ datapoints from all the MR curves of Fig. 9(a) moved on the $\rho (B,\theta ={0}^{\circ })$ curve. In result, all the $\rho (B,\theta )$ curves collapsed onto a single $\rho ({\varepsilon }_{\theta }B)\equiv \rho (B,\theta ={0}^{\circ })$ variation, as it is apparent from Fig. 9(b). The values ${\varepsilon }_{\theta }$ which were used in the MR data conversion are shown in the inset to Fig. 9(b). Interestingly, they can be almost perfectly approximated by the function

5 $${\varepsilon }_{\theta }={({\cos }^{2}\theta +{\gamma }^{-2}{\sin }^{2}\theta )}^{\mathrm{1/2}},$$

which represents a proportionality of ${\varepsilon }_{\theta }$ to the cross-section of ellipsoidal Fermi pocket with the parameter *γ* standing for the mass anisotropy^40^. From the fit of equation (5) to the data of LuSb (red solid line in the inset to Fig. 9(b)), we got $\gamma =4.55$. This value is larger than *γ* = 3.4 obtained for YSb in ref.^27^ and *γ* = 4 found for MoTe_2_ in ref.^41^, but smaller than $\gamma =4.762$ reported for WTe_2_ in ref.^39^. The so-obtained *γ* value slightly disagrees with ${k}_{{F}_{{\alpha }_{1}}}/{k}_{{F}_{\alpha }}\sim 3$, which also corresponds to the Fermi sheet anisotropy. The discrepancy may be due to a contribution from anisotropic features of the *δ* pocket, which was not taken into account in our analysis of the SdH effect.

**Figure 10:**
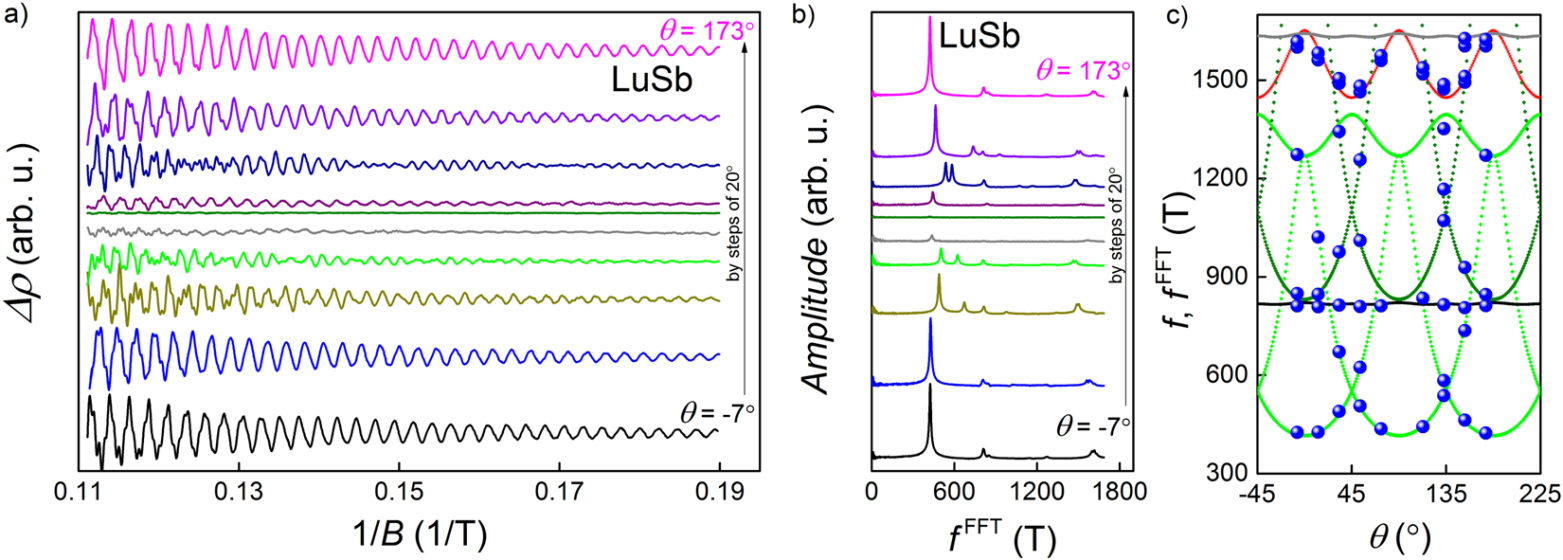
(**a**) Shubnikov-de Haas oscillations in LuSb measured at *T* = 2 K for different angles of applied magnetic field with respect to the direction of electric current. (**b**) Fast Fourier transform analysis of the SdH data presented in panel (a). (**c**) Comparison of the angular dependence of the SdH oscillations frequencies obtained from the FFT analysis, ${f}^{{\rm{FFT}}}$ (blue points) and from first-principles calculations (green and olive lines correspond respectively to the frequency due to the *α* Fermi pockets and the second harmonics of this frequency; black and grey lines refer respectively to the frequency and its second harmonics due to the *β* pocket; red line stands for the frequency due to the *δ* pocket).

In all the *ρ*(*B*) curves taken at different *θ* angles there were discernible SdH oscillations, which could be analyzed in the same way as it was described above. Figure 10(a) shows ${\rm{\Delta }}\rho$ as a function of inverted magnetic field, and Fig. 10(b) demonstrates their FFT evaluation. The angle dependences of the particular FFT frequencies are displayed in Fig. 10(c). The experimental data are compared on this figure with the results of the first-principles calculations. In order to get good agreement between the measured and calculated values of the SdH frequencies, the latter ones had to be multiplied by 0.855 (which does not influence the carrier compensation), such adjustment may be due to slightly enhanced lattice parameter (determined at room temperature) used in band structure calculations.

## Conclusions

Our comprehensive investigation of the magnetotransport behavior in single-crystalline LuSb revealed substantial MR effect, which is most likely a consequence of nearly perfect charge compensation in this material, as supported by the results of our first-principles electronic band structure calculations. The measured magnitude of MR slightly exceeded 3000%, but improving the sample quality would certainly allow to obtain significantly larger MR. The quality of our crystals permitted recording of clear Shubnikov–de Haas oscillations, which allowed for the determination of the Fermi surface topology in LuSb. The experimental SdH data were very well reproduced by our band structure calculations. Kohler scaling of magnetoresistance accounts very well for its temperature behavior, whereas the field-angle-dependent magnetoresistance could be scaled with the effective mass anisotropy perfectly agreeing with electronic structure and quantum oscillations analysis. The magnetotransport properties of LuSb could thus be well accounted for without invoking any topologically non-trivial electronic features.

This conclusion is in concert with the lack of band inversion and mixed *d* − *p* orbital texture in LuSb as well as findings from the most recent ARPES experiments^30^. Our results and previous reports on magnetotransport in lanthanum monopnictides show that studies of other rare-earth monopnictides might be crucial to elucidating the role of Dirac states in extreme magnetoresistance.

## Methods

Electronic structure calculations were performed with the all-electron general potential linearized augmented plane-wave (LAPW) method using the WIEN2k code^42^. Spin-orbit coupling was included as a second variational step, using scalar-relativistic eigenfunctions as the basis, after the initial calculation was converged to self-consistency. The Monkhorst–Pack special *k*-point scheme with 46 × 46 × 46 mesh was used in the first Brillouin zone sampling, and the cutoff parameter (${R}_{mt}{K}_{max}$) was set to 8. The modified Becke–Johnson potential^43^ was applied for improved estimates of the band gaps followed by regular self-consistent-field calculation using the GGA–PBE scheme^44^ for the exchange-correlation potential.

For the Fermi surface, the irreducible Brillouin zone was sampled by 20225 *k* points to ensure accurate determination of the Fermi level^45^. SdH frequencies were calculated using the Supercell K-space Extremal Area Finder tool^46^.

Single crystals of LuSb were grown from Sn flux in temperature regime selected accordingly to the binary phase diagram Lu-Sb^47^. The crystals were oriented and their quality was verified by backscattering Laue method using a Proto LAUE-COS system. Powdered crystals were examined by powder X-ray diffraction (XRD) employing a PANanalytical X’pert Pro diffractometer with Cu-K *α* radiation. The NaCl-type crystal structure was confirmed, with the cubic lattice parameter of 6.0577(1) Å, which is close to the literature value 6.0555 Å^18^. No impurity phases were observed on the XRD pattern. In addition, phase purity of the crystals and their chemical composition was checked by energy-dispersive X-ray spectroscopy on FEI scanning electron microscope equipped with an EDAX Genesis XM4 spectrometer.

Measurements of electrical resistivity, magnetoresistance and Hall resistivity were carried out in the temperature range from 0.7 to 300 K and in magnetic fields up to 9 T employing a Quantum Design PPMS-9 platform. Electrical contacts were made of silver wires attached to the rectangular-prism-shaped samples with silver epoxy, and additionally strengthened by spot welding. Electrical current was always flowing along [100] crystallographic direction.

### Data availability

The datasets analysed during the current study are available from the corresponding author on reasonable request.
